# A new example of intra­molecular C—H⋯Ni anagostic inter­actions: synthesis, crystal structure and Hirshfeld analysis of *cis*-bis­[4-methyl-2-(1,2,3,4-tetra­hydro­naphthalen-1-yl­idene)hydrazinecarbo­thio­amidato-κ^2^ *N* ^1^,*S*]nickel(II) di­methyl­formamide monosolvate

**DOI:** 10.1107/S2056989017007198

**Published:** 2017-05-19

**Authors:** Adriano Bof de Oliveira, Johannes Beck, Sônia Elizabeth Brown S. Mellone, Jörg Daniels

**Affiliations:** a Departamento de Química, Universidade Federal de Sergipe, Av. Marechal Rondon s/n, 49100-000 São Cristóvão-SE, Brazil; b Institut für Anorganische Chemie, Rheinische Friedrich-Wilhelms-Universität Bonn, Gerhard-Domagk-Strasse 1, D-53121 Bonn, Germany

**Keywords:** crystal structure, Ni—H anagostic inter­action, nickel-thio­semicarbazone *cis* complex

## Abstract

The reaction of Ni^II^ acetate tetra­hydrate with 4-methyl-2-(1,2,3,4-tetra­hydro­naphthalen-1-yl­idene)hydrazinecarbo­thio­amide in a 2:1 molar ratio and recrystallization from di­methyl­formamide yielded the title compound, [Ni(C_12_H_14_N_3_S)_2_]·C_3_H_7_NO. The ligands act as monoanionic κ^2^ *N* ^1^,*S*-donors, forming five-membered metallarings. The Ni^II^ ion is fourfold coordinated in a distorted square-planar *cis*-configuration, which is rather uncommon for mono­thio­semicarbazone complexes. Intra­molecular H⋯Ni *trans*-inter­actions are observed [H⋯Ni distances are 2.50 and 2.57 Å] and thus anagostic inter­actions can be suggested. The Hirshfeld surface analysis indicates that the major contributions for the crystal packing are H⋯H (66.6%), H⋯S (12.3%) and H⋯C (10.9%) inter­actions. In the crystal, the complex mol­ecules are linked by di­methyl­formamide solvent mol­ecules through N—H⋯O inter­actions into one-dimensional hydrogen-bonded polymers along [010].

## Abstract 2

The homoleptic nickel–thio­semicarbazonate complex shows structural features including an unusual *cis*-coordination and *trans*-anagostic Ni—H intra­molecular inter­actions. In the crystal, complex and DMF solvate mol­ecules build up a one-dimensional hydrogen-bonded polymer along [010].

## Chemical context   

One of the first reports on thio­semicarbazone chemistry can be traced back to the beginning of the 20th century in Germany (Freund & Schander, 1902 ▸). Initially, thio­semicarbazone derivatives were the products of the identification and characterization reactions of aldehydes and ketones, with thio­semicarbazide as reagent. In the 1940s it was reported that, in *in vitro* assays, thio­semicarbazones turned out to be very effective for the *Mycobacterium tuberculosis* growth inhibition (Domagk *et al.*, 1946 ▸) while the synthesis of thio­semicarbazone metal complexes had already been investigated by the early 1950s (Kuhn & Zilliken, 1952 ▸). As a result of the main fragment, *R*=N—N(H)—C(=S)—N*R*_2_, thio­semicarbazone derivatives have a wide range of coordination modes and applications in inorganic chemistry. The hydrazinic H atom can be easily removed and the negative charge is then delocalized over the C—N—N—C—S backbone, which enables chemical bonding with many different metal ions (Lobana *et al.*, 2009 ▸). However, a *cis* configuration of the ligated mol­ecules is a rather uncommon coordination mode for mono-thio­semicarbazones and, as far as we know, there is only one Ni^II^ mono-thio­semicarbazone complex reported in the literature, with *N*-phenyl-2-(1,2,3,4-tetra­hydro­naphthalen-1-yl­idene)hydrazinecarbothi­amide as ligand (for the ligand crystal structure, see: de Oliveira *et al.*, 2014*a* ▸; for the crystal structure of the complex, see: de Oliveira *et al.*, 2014*b* ▸). It can be suggested that the mol­ecular symmetry decreases from a *trans* to a *cis* configuration, possibly by loss of inversion symmetry at the central metal cation, which is compensated for by H⋯Ni intra­molecular inter­actions and hydrogen-bond formation with solvent mol­ecules. In general, H⋯metal ion inter­actions can show covalent or electrostatic character and are observed in some complexes with catalytic applications (Brookhart *et al.*, 2007 ▸). As part of our research on the synthesis and structural studies of thio­semicarbazone derivatives, we report herein a new solvated nickel homoleptic complex with the 4-methyl-2-(1,2,3,4-tetra­hydro­naphthalen-1-yl­idene)hydrazinecarbo­thio­amide ligand and di­methyl­formamide (DMF) as solvent.
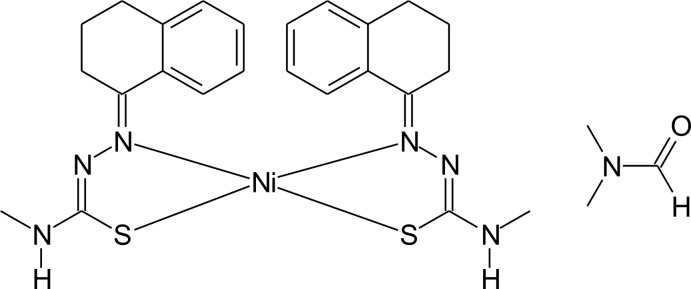


## Structural commentary   

**Figure 1:**
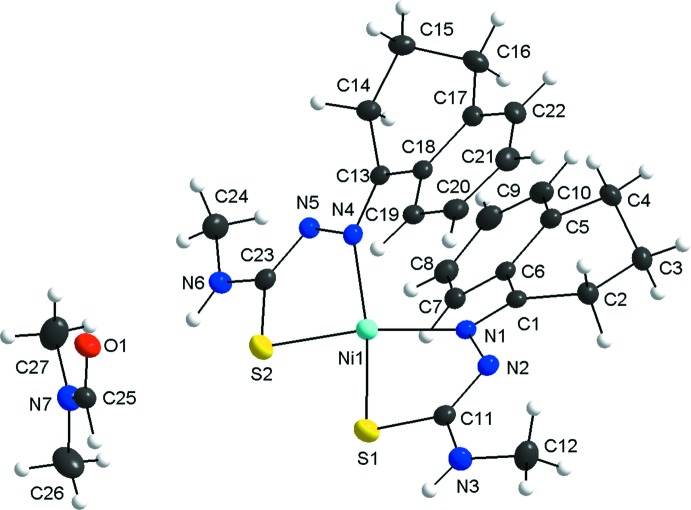
The mol­ecular structure of the title compound and the di­methyl­formamide solvate, with labelling and displacement ellipsoids drawn at the 40% probability level.

One mol­ecule of the title complex and one di­methyl­formamide solvate comprise the asymmetric unit. The Ni^II^ ion is fourfold coordinated in a distorted square-planar environment by two chelating thio­semicarbazonate ligands (Fig. 1 ▸). The maximum deviation from the Ni1/S1/S2/N1/N4 mean plane amounts to 0.1705 (16) Å for N1. The S1—Ni1—N4 and S2—Ni1—N1 bond angles are 169.42 (5) and 168.38 (5)°, respectively. The distortion along the *trans-*donor atoms confirms the deviation of the coordination sphere from ideal values. Both non-aromatic rings of the tetra­lone entities have an envelope conformation with maximum deviations from the mean plane of the non–H atoms of 0.3539 (15) Å for C3 and of 0.3685 (15) Å for C15. The two ligands are deprotonated with the negative charge delocalized over the C—N—N—C—S entity, as suggested by their inter­mediate bond lengths and supported by the *sp* ^2^-hybridization for C1, C13, N1, C11, C23 and N4. The imine and thio­amide C—N distances indicate considerable double-bond character, while the C—S distance is consistent with mainly single-bond character. The change of the bond lengths is a key feature to distinguish free, *i.e.* non-coordinating, and coordinating thio­semicarbazones. For the title compound these distances (values given in Å) are C1—N1 = 1.306 (2), N1—N2 = 1.408 (2), N2—C11 = 1.307 (2) and C11—S1 = 1.752 (2) for one ligand and C13—N4 = 1.303 (2), N4—N5 = 1.409 (2), N5—C23 = 1.303 (2) and C23—S2 = 1.7573 (19) for the other one.

**Figure 2:**
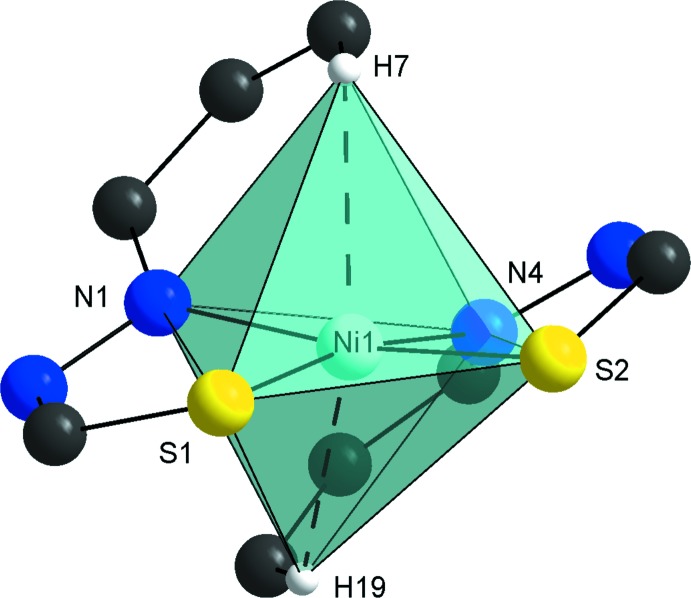
Graphical representation of the metal ion coordination environment, showing the H7⋯Ni1 and H19⋯Ni1 anagostic inter­actions as dashed lines. The figure is simplified for clarity.

The title complex shows two remarkable structural features, namely a *cis* coordination mode, which is rather uncommon for mono-thio­semicarbazone ligands, as well as two positioned *trans* H⋯Ni anagostic inter­actions (Fig. 2 ▸, Table 1 ▸). The H7⋯Ni1 and H19⋯Ni1 distances are 2.50 and 2.57 Å, being shorter than the sum of the van der Waals radii for H and Ni (2.73 Å; Bondi, 1964 ▸; Rowland & Taylor, 1996 ▸), in order that electrostactic inter­actions can be assigned. For an agostic inter­action, that involves a covalent or a three-center, two-electron bond, an H⋯metal distance of at least 2.3 Å is required. The C7—H7⋯Ni1 and C19—H19⋯Ni1 angles are 120.1 and 119.7°, being in agreement with literature data for another nickel complex with anagostic inter­actions (de Oliveira *et al.*, 2014*b* ▸).

## Supra­molecular features and Hirshfeld surface analysis   

**Figure 3:**
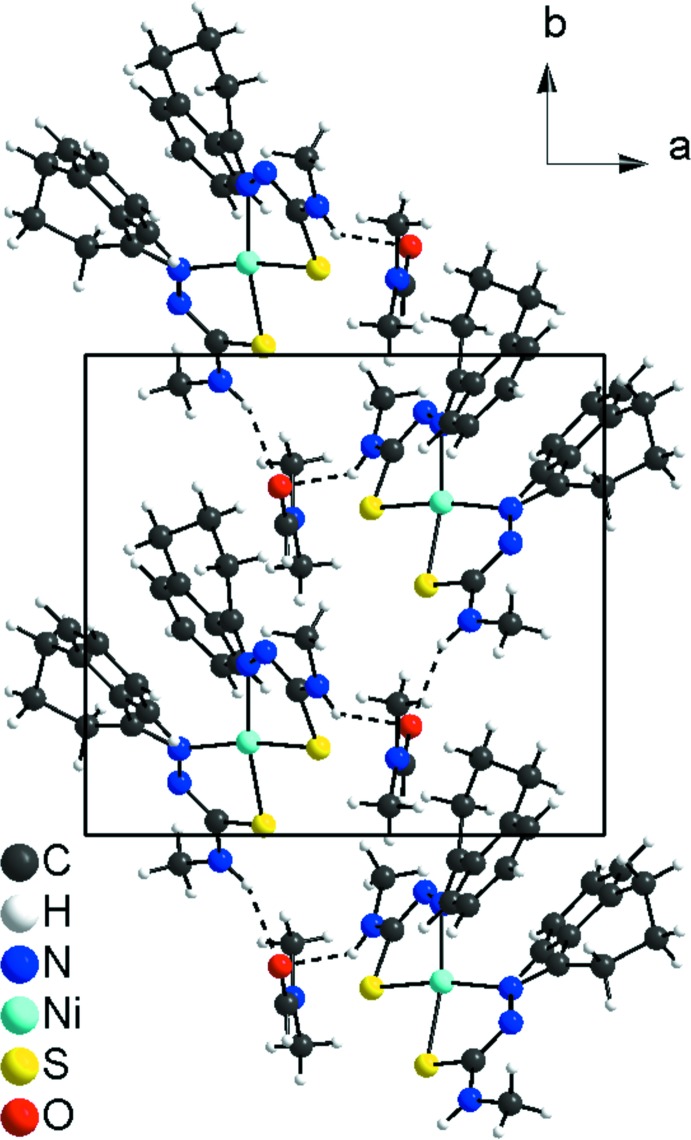
Section of the crystal structure of the title compound viewed along [001], with hydrogen bonds shown as dashed lines (for details, see: Table 1 ▸). The figure is simplified for clarity.

In the crystal, the coordination entities are linked by DMF solvate mol­ecules through N—H⋯O inter­actions. The DMF-oxygen atoms are hydrogen-bond acceptors, forming a bridg­ing structure between two N—H⋯O arrangements: N6—H6⋯O1 and N3—H3⋯O1^i^ [symmetry code: (i) −*x* + 1, *y* − 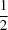, −*z* + 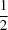]. The mol­ecules are linked into one-dimensional hydrogen-bonded polymers along [010] (Fig. 3 ▸, Table 1 ▸). Additional C—H⋯O inter­actions are also present (Table 1 ▸).

**Figure 4:**
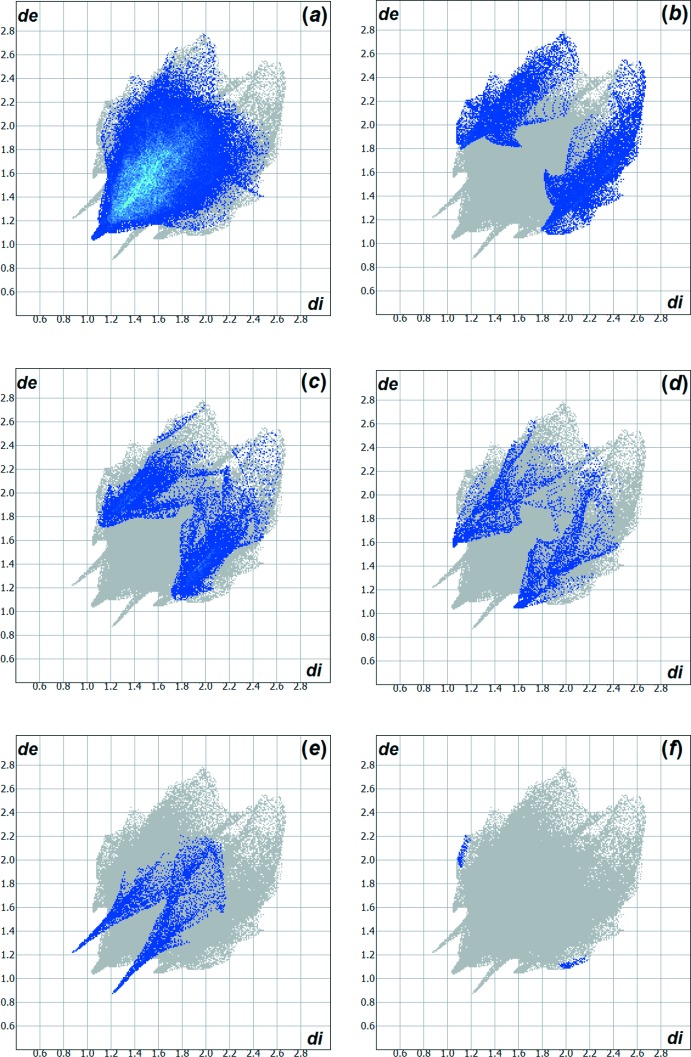
Graphical representation of the two-dimensional Hirshfeld surface fingerprint plots for the inter­actions in the crystal structure of the title compound. The contacts are drawn in detail (cyan dots) and the contributions to the crystal packing amount to: (*a*) H⋯H = 66.6%, (*b*) H⋯S = 12.3%, (*c*) H⋯C = 10.9%, (*d*) H⋯N = 4.5%, (*e*) H⋯O = 4.0% and (*f*) H⋯Ni = 0.2%. The *d*_e_ (*y* axis) and *d*_i_ (*x* axis) values are the closest external and inter­nal distances (values in Å) from given points on the Hirshfeld surface contacts.

Hirshfeld (1977 ▸) analysis of the crystal structure suggests that the inter­molecular H⋯H inter­actions contribute 66.6% to the crystal packing, the H⋯S inter­actions 12.3% and the H⋯C inter­actions 10.9%. Other important inter­molecular contacts for the cohesion of the mol­ecules are H⋯N = 4.5% and H⋯O = 4.0%. The weak H⋯Ni inter­actions contribute by 0.20% to the crystal structure. All contributions to the crystal cohesion are shown as two-dimensional Hirshfeld surface fingerprint plots with cyan dots (Wolff *et al.*, 2012 ▸). The *d*_e_ (*y* axis) and *d*_i_ (*x* axis) values are the closest external and inter­nal distances (values in Å) from given points on the Hirshfeld surface contacts (Fig. 4 ▸).

## Comparison with a related structure   

**Figure 5:**
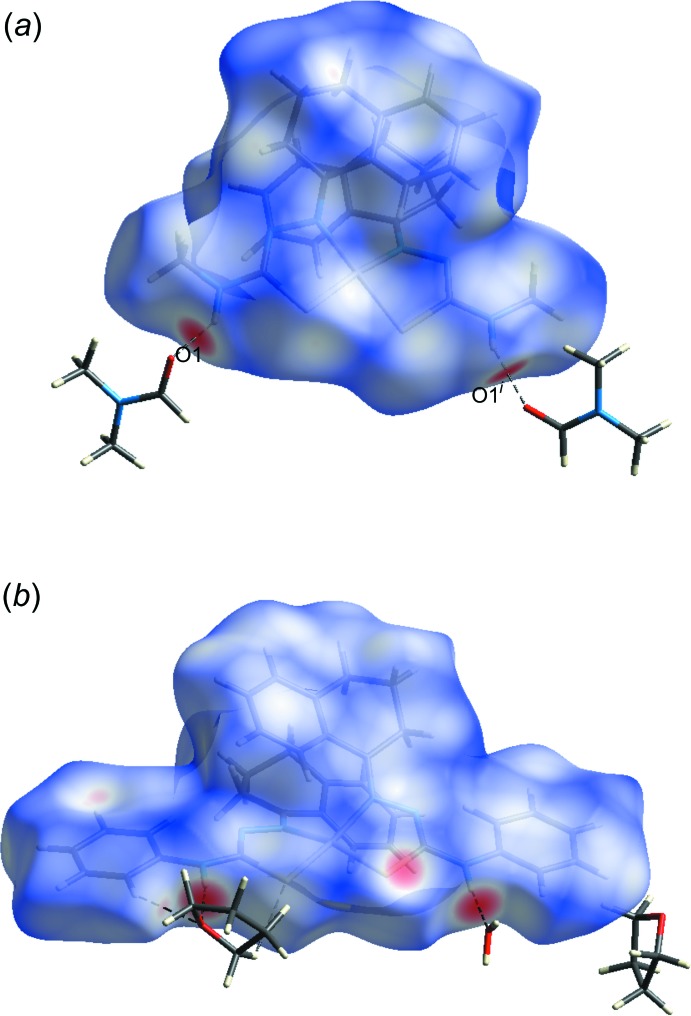
The Hirshfeld surface graphical representation (*d*_norm_) for: (*a*) the asymmetric unit of the title compound and (*b*) the asymmetric unit of the comparison compound, bis­{*cis*-(2-(1,2,3,4-tetra­hydro­naphthalen-1-yl­idene)-4-phenyl-hydrazinecarbo­thio­amidate-κ^2^ *N* ^1^,*S*)}nickel(II) monohydrate bis­(tetra­hydro­furane) solvate (de Oliveira *et al.* 2014*b* ▸). The surface regions with the strongest inter­molecular inter­actions are drawn in magenta. The figure is simplified for clarity. [Symmetry code: (i) −*x* + 1, *y* − 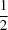, −*z* + 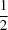.]

For comparison with the title compound, a literature search revealed only one crystal structure of an Ni^II^–mono­thio­semicarbazone complex with *cis* configuration, *viz*. bis­{*cis*-(2-(1,2,3,4-tetra­hydro­naphthalen-1-yl­idene)-4-phenyl-hydrazine­carbo­thio­amidate-κ^2^ *N* ^1^,*S*)}nickel(II) monohydrate bis­(tetra­hydro­furane) solvate (de Oliveira *et al.* 2014*b* ▸). The graphical representation of the Hirshfeld surface was performed for the two complexes and suggests, represented in magenta colour, the locations of the strongest inter­molecular contacts (Fig. 5 ▸). Both structures have the same main fragment for the ligand, the α-tetra­lone-thio­semicarbazone, anagostic H⋯Ni intra­molecular inter­actions and hydrogen bonding with the solvate mol­ecules, suggesting the stabilization of the crystal packing, since the *cis* configuration implies a symmetry decrease with loss of the inversion center and appears to be energetically unfavourable.

## Synthesis and crystallization   

Starting materials were commercially available and were used without further purification. The synthesis of the ligand was adapted from a procedure reported previously (Freund & Schander, 1902 ▸) with 1-tetra­lone and 4-methyl­thio­semicarbazide. 2-(1,2,3,4-Tetra­hydro­naphthalen-1-yl­idene)-4-methyl-hydrazinecarbo­thio­amide was dissolved in tetra­hydro­furan (THF; 2 mmol / 40 ml) with stirring maintained for 30 min until the solution turned yellow. At the same time, a green solution of nickel acetate tetra­hydrate in THF (1 mmol/40 ml) was prepared under continuous stirring. A dark coloured mixture of both solutions was maintained with stirring at room temperature for 6 h. A crude dark red material was obtained by evaporation of the solvent. Dark red crystals of the complex, suitable for X-ray analysis, were obtained by recrystallization of the solid from a di­methyl­formamide solution.

## Refinement   

Crystal data, data collection and structure refinement details are summarized in Table 2 ▸. All H atoms were located in difference maps but were positioned with idealized geometry and were refined using a riding model with *U*_iso_(H) = 1.2*U*_eq_(C and N) for the *sp* ^2^–hybridized DMF C atom, the aromatic and the secondary C atoms, and for all N atoms, and with *U*_iso_(H) = 1.5*U*_eq_(C) for the methyl C atoms. The bond lengths (values given in Å) are: C—H = 0.99 for –CH_2_– fragments, C—H = 0.98 for CH_3_– fragments, C—H = 0.95 for aromatic groups and the *sp* ^2^-hybridized DMF C atom; N—H = 0.88 for all N atoms.

## Supplementary Material

Crystal structure: contains datablock(s) I. DOI: 10.1107/S2056989017007198/wm5389sup1.cif

Structure factors: contains datablock(s) I. DOI: 10.1107/S2056989017007198/wm5389Isup2.hkl

CCDC reference: 1550129

Additional supporting information:  crystallographic information; 3D view; checkCIF report

## Figures and Tables

**Table 1:** Hydrogen-bond geometry (Å, °)

*D*—H⋯*A*	*D*—H	H⋯*A*	*D*⋯*A*	*D*—H⋯*A*
N3—H3⋯O1^i^	0.88	2.18	2.979 (2)	151
N6—H6⋯O1	0.88	2.14	2.875 (2)	140
C7—H7⋯Ni1	0.95	2.50	3.0831 (19)	120
C19—H19⋯Ni1	0.95	2.57	3.1480 (19)	120

Symmetry code: (i) 
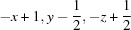
.

**Table 2:** Experimental details

Crystal data	
Chemical formula	[Ni(C_12_H_14_N_3_S)_2_]·C_3_H_7_NO
*M*_r_	596.45
Crystal system, space group	Monoclinic, *P*2_1_/*c*
Temperature (K)	123
*a*, *b*, *c* (Å)	12.5864 (3), 11.6273 (3), 19.1271 (5)
β (°)	90.529 (1)
*V* (Å^3^)	2799.05 (12)
*Z*	4
Radiation type	Mo *K*α
μ (mm^−1^)	0.88
Crystal size (mm)	0.33 × 0.14 × 0.02

Data collection	
Diffractometer	Nonius Kappa CCD area detector
Absorption correction	Multi-scan (Blessing, 1995 ▸)
*T*_min_, *T*_max_	0.761, 0.981
No. of measured, independent and observed [*I* > 2σ(*I*)] reflections	46078, 6368, 4870
*R*_int_	0.057
(sin θ/λ)_max_ (Å^−1^)	0.649

Refinement	
*R*[*F* ^2^ > 2σ(*F* ^2^)], *wR*(*F* ^2^), *S*	0.034, 0.081, 1.03
No. of reflections	6368
No. of parameters	347
H-atom treatment	H-atom parameters constrained
Δρ_max_, Δρ_min_ (e Å^−3^)	0.28, −0.35

Computer programs: *COLLECT* (Nonius, 1998 ▸), *DENZO* and *SCALEPACK* (Otwinowski & Minor, 1997 ▸), *SHELXS97* (Sheldrick, 2008 ▸), *SHELXL2016* (Sheldrick, 2015 ▸), *WinGX* (Farrugia, 2012 ▸), *DIAMOND* (Brandenburg, 2006 ▸), *Crystal Explorer* (Wolff *et al.*, 2012 ▸), *publCIF* (Westrip, 2010 ▸) and *enCIFer* (Allen *et al.*, 2004 ▸).

## supplementary crystallographic information

### Crystal data

**Table:**

[Ni(C_12_H_14_N_3_S)_2_]·C_3_H_7_NO	*F*(000) = 1256
*M**_r_* = 596.45	*D*_x_ = 1.415 Mg m^−^^3^
Monoclinic, *P*2_1_/*c*	Mo *K*α radiation, λ = 0.71073 Å
*a* = 12.5864 (3) Å	Cell parameters from 105836 reflections
*b* = 11.6273 (3) Å	θ = 2.9–27.5°
*c* = 19.1271 (5) Å	µ = 0.88 mm^−^^1^
β = 90.529 (1)°	*T* = 123 K
*V* = 2799.05 (12) Å^3^	Plate, dark red
*Z* = 4	0.33 × 0.14 × 0.02 mm

### Data collection

**Table:**

Nonius Kappa CCD area detector diffractometer	6368 independent reflections
Radiation source: fine-focus sealed X-ray tube, Enraf–Nonius FR590	4870 reflections with *I* > 2σ(*I*)
Detector resolution: 9 pixels mm^-1^	*R*_int_ = 0.057
CCD area detector scans	θ_max_ = 27.5°, θ_min_ = 3.2°
Absorption correction: multi-scan (Blessing, 1995)	*h* = −16→16
*T*_min_ = 0.761, *T*_max_ = 0.981	*k* = −15→15
46078 measured reflections	*l* = −24→24

### Refinement

**Table:**

Refinement on *F*^2^	Primary atom site location: structure-invariant direct methods
Least-squares matrix: full	Secondary atom site location: difference Fourier map
*R*[*F*^2^ > 2σ(*F*^2^)] = 0.034	Hydrogen site location: inferred from neighbouring sites
*wR*(*F*^2^) = 0.081	H-atom parameters constrained
*S* = 1.03	*w* = 1/[σ^2^(*F*_o_^2^) + (0.0352*P*)^2^ + 1.1854*P*] where *P* = (*F*_o_^2^ + 2*F*_c_^2^)/3
6368 reflections	(Δ/σ)_max_ = 0.001
347 parameters	Δρ_max_ = 0.28 e Å^−^^3^
0 restraints	Δρ_min_ = −0.35 e Å^−^^3^

### Special details

**Table:**

Geometry. All esds (except the esd in the dihedral angle between two l.s. planes) are estimated using the full covariance matrix. The cell esds are taken into account individually in the estimation of esds in distances, angles and torsion angles; correlations between esds in cell parameters are only used when they are defined by crystal symmetry. An approximate (isotropic) treatment of cell esds is used for estimating esds involving l.s. planes.

### Fractional atomic coordinates and isotropic or equivalent isotropic displacement parameters (Å^2^)

**Table:**

	*x*	*y*	*z*	*U*_iso_*/*U*_eq_	
C1	0.08922 (15)	0.22945 (15)	0.16633 (10)	0.0211 (4)	
C2	0.00261 (15)	0.22610 (17)	0.11111 (11)	0.0263 (4)	
H2A	−0.012932	0.144831	0.099439	0.032*	
H2B	0.028999	0.263837	0.068247	0.032*	
C3	−0.10022 (16)	0.28467 (17)	0.13303 (11)	0.0276 (5)	
H3A	−0.145309	0.299214	0.091278	0.033*	
H3B	−0.139887	0.233640	0.164970	0.033*	
C4	−0.07562 (16)	0.39797 (17)	0.16967 (11)	0.0286 (5)	
H4A	−0.034965	0.448847	0.138186	0.034*	
H4B	−0.142564	0.437423	0.182052	0.034*	
C5	−0.01161 (15)	0.37338 (16)	0.23472 (11)	0.0246 (4)	
C6	0.06834 (15)	0.28808 (15)	0.23276 (10)	0.0214 (4)	
C7	0.11941 (15)	0.25627 (16)	0.29524 (10)	0.0238 (4)	
H7	0.169921	0.195566	0.294964	0.029*	
C8	0.09729 (16)	0.31204 (17)	0.35734 (11)	0.0280 (4)	
H8	0.133779	0.290933	0.399106	0.034*	
C9	0.02163 (16)	0.39893 (18)	0.35845 (12)	0.0308 (5)	
H9	0.007412	0.438515	0.400793	0.037*	
C10	−0.03313 (16)	0.42788 (17)	0.29776 (12)	0.0293 (5)	
H10	−0.086231	0.485872	0.299236	0.035*	
C11	0.25278 (15)	0.02857 (16)	0.10116 (10)	0.0233 (4)	
C12	0.18174 (17)	−0.0606 (2)	−0.00542 (12)	0.0352 (5)	
H12A	0.111995	−0.080067	0.013631	0.053*	
H12B	0.203560	−0.120404	−0.038434	0.053*	
H12C	0.177475	0.013501	−0.029660	0.053*	
C13	0.28111 (14)	0.44637 (15)	0.19613 (10)	0.0196 (4)	
C14	0.27262 (16)	0.56078 (16)	0.23328 (10)	0.0252 (4)	
H14A	0.342572	0.580051	0.254263	0.030*	
H14B	0.221089	0.553388	0.271814	0.030*	
C15	0.23767 (17)	0.65918 (16)	0.18558 (11)	0.0284 (5)	
H15A	0.216582	0.726228	0.214176	0.034*	
H15B	0.297515	0.682696	0.155546	0.034*	
C16	0.14426 (17)	0.62064 (17)	0.14013 (12)	0.0315 (5)	
H16A	0.084832	0.595538	0.170053	0.038*	
H16B	0.119187	0.685479	0.110766	0.038*	
C17	0.17939 (15)	0.52246 (16)	0.09428 (11)	0.0244 (4)	
C18	0.25161 (14)	0.44030 (15)	0.12149 (10)	0.0219 (4)	
C19	0.29536 (15)	0.35877 (16)	0.07606 (10)	0.0233 (4)	
H19	0.347667	0.306746	0.093171	0.028*	
C20	0.26353 (16)	0.35283 (17)	0.00660 (11)	0.0281 (5)	
H20	0.293256	0.296584	−0.023476	0.034*	
C21	0.18816 (17)	0.42932 (18)	−0.01872 (11)	0.0313 (5)	
H21	0.164134	0.423931	−0.065837	0.038*	
C22	0.14775 (16)	0.51388 (17)	0.02480 (11)	0.0288 (5)	
H22	0.097302	0.567064	0.006610	0.035*	
C23	0.40915 (15)	0.30161 (16)	0.32372 (10)	0.0223 (4)	
C24	0.42580 (18)	0.41030 (18)	0.43256 (11)	0.0325 (5)	
H24A	0.461284	0.477466	0.412455	0.049*	
H24B	0.453003	0.396954	0.480046	0.049*	
H24C	0.349070	0.424227	0.434154	0.049*	
N1	0.18025 (12)	0.17918 (12)	0.15512 (8)	0.0203 (3)	
N2	0.18018 (12)	0.10848 (13)	0.09533 (8)	0.0225 (3)	
N3	0.25886 (13)	−0.05314 (14)	0.05102 (9)	0.0283 (4)	
H3	0.311231	−0.103222	0.052768	0.034*	
N4	0.31278 (12)	0.35542 (13)	0.23026 (8)	0.0208 (3)	
N5	0.34549 (12)	0.38054 (13)	0.29923 (8)	0.0224 (3)	
N6	0.44659 (13)	0.31004 (14)	0.38969 (9)	0.0258 (4)	
H6	0.484635	0.253290	0.407241	0.031*	
Ni1	0.31331 (2)	0.19296 (2)	0.20574 (2)	0.01982 (8)	
S1	0.34284 (4)	0.01916 (4)	0.17128 (3)	0.02631 (12)	
S2	0.44889 (4)	0.18089 (4)	0.27525 (3)	0.02496 (12)	
C25	0.61277 (16)	0.14667 (18)	0.51104 (11)	0.0279 (5)	
H25	0.611205	0.070634	0.492874	0.034*	
C26	0.5851 (2)	0.0599 (2)	0.62485 (14)	0.0474 (6)	
H26A	0.586504	−0.010812	0.597040	0.071*	
H26B	0.516179	0.066792	0.647931	0.071*	
H26C	0.641814	0.057435	0.660262	0.071*	
C27	0.60202 (19)	0.2724 (2)	0.61103 (13)	0.0431 (6)	
H27A	0.615740	0.330474	0.575109	0.065*	
H27B	0.657953	0.275813	0.646952	0.065*	
H27C	0.532977	0.287397	0.632476	0.065*	
N7	0.60110 (14)	0.15880 (15)	0.57921 (9)	0.0304 (4)	
O1	0.62554 (12)	0.22590 (12)	0.46895 (8)	0.0328 (3)	

### Atomic displacement parameters (Å^2^)

**Table:**

	*U*^11^	*U*^22^	*U*^33^	*U*^12^	*U*^13^	*U*^23^
C1	0.0211 (10)	0.0162 (8)	0.0260 (11)	−0.0018 (7)	0.0004 (8)	0.0019 (8)
C2	0.0247 (10)	0.0255 (10)	0.0285 (12)	0.0013 (8)	−0.0029 (9)	−0.0011 (8)
C3	0.0223 (10)	0.0293 (11)	0.0312 (12)	0.0011 (8)	−0.0033 (9)	0.0016 (9)
C4	0.0235 (10)	0.0248 (10)	0.0374 (13)	0.0051 (8)	−0.0025 (9)	0.0029 (9)
C5	0.0209 (10)	0.0195 (9)	0.0333 (12)	−0.0013 (8)	0.0021 (8)	−0.0005 (8)
C6	0.0194 (9)	0.0178 (9)	0.0271 (11)	−0.0031 (7)	0.0016 (8)	−0.0004 (8)
C7	0.0201 (10)	0.0228 (9)	0.0287 (11)	−0.0009 (8)	0.0024 (8)	0.0009 (8)
C8	0.0306 (11)	0.0283 (10)	0.0250 (11)	−0.0052 (9)	0.0010 (9)	−0.0006 (9)
C9	0.0315 (11)	0.0296 (11)	0.0314 (12)	−0.0041 (9)	0.0080 (9)	−0.0088 (9)
C10	0.0242 (10)	0.0236 (10)	0.0403 (13)	0.0004 (8)	0.0065 (9)	−0.0034 (9)
C11	0.0236 (10)	0.0209 (9)	0.0256 (11)	−0.0023 (8)	0.0027 (8)	0.0001 (8)
C12	0.0311 (11)	0.0421 (12)	0.0322 (13)	−0.0016 (10)	−0.0005 (9)	−0.0130 (10)
C13	0.0177 (9)	0.0191 (9)	0.0219 (10)	−0.0024 (7)	0.0034 (7)	−0.0003 (7)
C14	0.0288 (10)	0.0208 (9)	0.0259 (11)	0.0011 (8)	0.0033 (8)	−0.0017 (8)
C15	0.0349 (12)	0.0202 (9)	0.0300 (12)	0.0023 (8)	0.0048 (9)	−0.0010 (8)
C16	0.0313 (11)	0.0266 (10)	0.0365 (13)	0.0077 (9)	0.0020 (9)	0.0040 (9)
C17	0.0206 (10)	0.0221 (9)	0.0305 (12)	−0.0016 (8)	0.0008 (8)	0.0030 (8)
C18	0.0202 (9)	0.0191 (9)	0.0265 (11)	−0.0029 (7)	0.0002 (8)	0.0034 (8)
C19	0.0249 (10)	0.0195 (9)	0.0254 (11)	−0.0013 (8)	−0.0008 (8)	0.0023 (8)
C20	0.0341 (11)	0.0226 (10)	0.0278 (12)	−0.0018 (9)	−0.0004 (9)	−0.0004 (8)
C21	0.0374 (12)	0.0297 (11)	0.0266 (12)	−0.0047 (9)	−0.0077 (9)	0.0037 (9)
C22	0.0280 (11)	0.0264 (10)	0.0319 (12)	0.0003 (8)	−0.0066 (9)	0.0072 (9)
C23	0.0216 (9)	0.0228 (9)	0.0225 (10)	−0.0040 (8)	0.0030 (8)	−0.0009 (8)
C24	0.0387 (12)	0.0327 (11)	0.0261 (12)	0.0000 (10)	−0.0045 (9)	−0.0062 (9)
N1	0.0207 (8)	0.0175 (7)	0.0226 (9)	−0.0006 (6)	0.0012 (7)	−0.0013 (6)
N2	0.0243 (8)	0.0207 (8)	0.0225 (9)	−0.0013 (7)	0.0000 (7)	−0.0036 (7)
N3	0.0294 (9)	0.0260 (9)	0.0296 (10)	0.0039 (7)	−0.0008 (8)	−0.0077 (7)
N4	0.0195 (8)	0.0218 (8)	0.0210 (9)	−0.0020 (6)	0.0001 (6)	−0.0007 (7)
N5	0.0238 (8)	0.0221 (8)	0.0211 (9)	0.0000 (7)	−0.0022 (7)	−0.0006 (6)
N6	0.0308 (9)	0.0244 (8)	0.0220 (9)	0.0015 (7)	−0.0042 (7)	0.0007 (7)
Ni1	0.01978 (13)	0.01747 (12)	0.02217 (14)	0.00112 (9)	−0.00106 (10)	−0.00098 (10)
S1	0.0284 (3)	0.0210 (2)	0.0294 (3)	0.0053 (2)	−0.0037 (2)	−0.0036 (2)
S2	0.0247 (3)	0.0250 (2)	0.0250 (3)	0.0051 (2)	−0.0034 (2)	−0.0018 (2)
C25	0.0265 (11)	0.0236 (10)	0.0336 (12)	0.0020 (8)	−0.0022 (9)	0.0000 (9)
C26	0.0446 (14)	0.0546 (15)	0.0431 (15)	0.0054 (12)	0.0074 (12)	0.0223 (12)
C27	0.0392 (13)	0.0516 (14)	0.0385 (14)	−0.0030 (11)	0.0065 (11)	−0.0151 (11)
N7	0.0290 (9)	0.0337 (9)	0.0285 (10)	0.0015 (8)	0.0031 (8)	0.0028 (8)
O1	0.0356 (8)	0.0314 (8)	0.0314 (9)	0.0029 (6)	−0.0007 (7)	0.0081 (7)

### Geometric parameters (Å, º)

**Table:**

C1—N1	1.306 (2)	C16—H16A	0.9900
C1—C6	1.468 (3)	C16—H16B	0.9900
C1—C2	1.511 (3)	C17—C22	1.387 (3)
C2—C3	1.525 (3)	C17—C18	1.415 (3)
C2—H2A	0.9900	C18—C19	1.402 (3)
C2—H2B	0.9900	C19—C20	1.386 (3)
C3—C4	1.523 (3)	C19—H19	0.9500
C3—H3A	0.9900	C20—C21	1.385 (3)
C3—H3B	0.9900	C20—H20	0.9500
C4—C5	1.503 (3)	C21—C22	1.388 (3)
C4—H4A	0.9900	C21—H21	0.9500
C4—H4B	0.9900	C22—H22	0.9500
C5—C10	1.391 (3)	C23—N5	1.303 (2)
C5—C6	1.414 (3)	C23—N6	1.347 (3)
C6—C7	1.401 (3)	C23—S2	1.7573 (19)
C7—C8	1.384 (3)	C24—N6	1.450 (3)
C7—H7	0.9500	C24—H24A	0.9800
C8—C9	1.389 (3)	C24—H24B	0.9800
C8—H8	0.9500	C24—H24C	0.9800
C9—C10	1.386 (3)	N1—N2	1.408 (2)
C9—H9	0.9500	N1—Ni1	1.9334 (16)
C10—H10	0.9500	N3—H3	0.8800
C11—N2	1.307 (2)	N4—N5	1.409 (2)
C11—N3	1.352 (2)	N4—Ni1	1.9463 (16)
C11—S1	1.752 (2)	N6—H6	0.8800
C12—N3	1.448 (3)	Ni1—S2	2.1581 (5)
C12—H12A	0.9800	Ni1—S1	2.1589 (5)
C12—H12B	0.9800	C25—O1	1.235 (2)
C12—H12C	0.9800	C25—N7	1.321 (3)
C13—N4	1.303 (2)	C25—H25	0.9500
C13—C18	1.474 (3)	C26—N7	1.458 (3)
C13—C14	1.512 (3)	C26—H26A	0.9800
C14—C15	1.526 (3)	C26—H26B	0.9800
C14—H14A	0.9900	C26—H26C	0.9800
C14—H14B	0.9900	C27—N7	1.454 (3)
C15—C16	1.523 (3)	C27—H27A	0.9800
C15—H15A	0.9900	C27—H27B	0.9800
C15—H15B	0.9900	C27—H27C	0.9800
C16—C17	1.508 (3)		
			
N1—C1—C6	120.96 (17)	H16A—C16—H16B	108.3
N1—C1—C2	120.13 (17)	C22—C17—C18	118.80 (18)
C6—C1—C2	118.90 (16)	C22—C17—C16	121.89 (18)
C1—C2—C3	113.89 (17)	C18—C17—C16	119.21 (18)
C1—C2—H2A	108.8	C19—C18—C17	118.90 (18)
C3—C2—H2A	108.8	C19—C18—C13	122.41 (17)
C1—C2—H2B	108.8	C17—C18—C13	118.65 (17)
C3—C2—H2B	108.8	C20—C19—C18	121.08 (18)
H2A—C2—H2B	107.7	C20—C19—H19	119.5
C4—C3—C2	110.09 (16)	C18—C19—H19	119.5
C4—C3—H3A	109.6	C21—C20—C19	119.63 (19)
C2—C3—H3A	109.6	C21—C20—H20	120.2
C4—C3—H3B	109.6	C19—C20—H20	120.2
C2—C3—H3B	109.6	C20—C21—C22	119.9 (2)
H3A—C3—H3B	108.2	C20—C21—H21	120.0
C5—C4—C3	108.75 (16)	C22—C21—H21	120.0
C5—C4—H4A	109.9	C17—C22—C21	121.46 (19)
C3—C4—H4A	109.9	C17—C22—H22	119.3
C5—C4—H4B	109.9	C21—C22—H22	119.3
C3—C4—H4B	109.9	N5—C23—N6	119.63 (17)
H4A—C4—H4B	108.3	N5—C23—S2	123.38 (15)
C10—C5—C6	119.14 (19)	N6—C23—S2	116.98 (14)
C10—C5—C4	121.57 (18)	N6—C24—H24A	109.5
C6—C5—C4	119.19 (18)	N6—C24—H24B	109.5
C7—C6—C5	118.92 (18)	H24A—C24—H24B	109.5
C7—C6—C1	122.10 (17)	N6—C24—H24C	109.5
C5—C6—C1	118.81 (18)	H24A—C24—H24C	109.5
C8—C7—C6	120.93 (18)	H24B—C24—H24C	109.5
C8—C7—H7	119.5	C1—N1—N2	113.62 (16)
C6—C7—H7	119.5	C1—N1—Ni1	129.64 (13)
C7—C8—C9	119.8 (2)	N2—N1—Ni1	116.69 (11)
C7—C8—H8	120.1	C11—N2—N1	110.51 (16)
C9—C8—H8	120.1	C11—N3—C12	121.94 (17)
C10—C9—C8	119.98 (19)	C11—N3—H3	119.0
C10—C9—H9	120.0	C12—N3—H3	119.0
C8—C9—H9	120.0	C13—N4—N5	112.71 (15)
C9—C10—C5	121.07 (19)	C13—N4—Ni1	131.95 (13)
C9—C10—H10	119.5	N5—N4—Ni1	115.17 (11)
C5—C10—H10	119.5	C23—N5—N4	111.34 (15)
N2—C11—N3	118.87 (18)	C23—N6—C24	121.66 (17)
N2—C11—S1	123.82 (15)	C23—N6—H6	119.2
N3—C11—S1	117.30 (14)	C24—N6—H6	119.2
N3—C12—H12A	109.5	N1—Ni1—N4	101.32 (6)
N3—C12—H12B	109.5	N1—Ni1—S2	168.38 (5)
H12A—C12—H12B	109.5	N4—Ni1—S2	85.36 (5)
N3—C12—H12C	109.5	N1—Ni1—S1	85.43 (5)
H12A—C12—H12C	109.5	N4—Ni1—S1	169.42 (5)
H12B—C12—H12C	109.5	S2—Ni1—S1	89.42 (2)
N4—C13—C18	121.30 (16)	C11—S1—Ni1	93.63 (6)
N4—C13—C14	120.07 (17)	C23—S2—Ni1	92.62 (7)
C18—C13—C14	118.62 (16)	O1—C25—N7	125.5 (2)
C13—C14—C15	113.58 (17)	O1—C25—H25	117.3
C13—C14—H14A	108.9	N7—C25—H25	117.3
C15—C14—H14A	108.9	N7—C26—H26A	109.5
C13—C14—H14B	108.9	N7—C26—H26B	109.5
C15—C14—H14B	108.9	H26A—C26—H26B	109.5
H14A—C14—H14B	107.7	N7—C26—H26C	109.5
C16—C15—C14	109.72 (16)	H26A—C26—H26C	109.5
C16—C15—H15A	109.7	H26B—C26—H26C	109.5
C14—C15—H15A	109.7	N7—C27—H27A	109.5
C16—C15—H15B	109.7	N7—C27—H27B	109.5
C14—C15—H15B	109.7	H27A—C27—H27B	109.5
H15A—C15—H15B	108.2	N7—C27—H27C	109.5
C17—C16—C15	109.00 (16)	H27A—C27—H27C	109.5
C17—C16—H16A	109.9	H27B—C27—H27C	109.5
C15—C16—H16A	109.9	C25—N7—C27	120.63 (19)
C17—C16—H16B	109.9	C25—N7—C26	121.6 (2)
C15—C16—H16B	109.9	C27—N7—C26	117.8 (2)
			
N1—C1—C2—C3	177.81 (17)	C14—C13—C18—C17	−24.9 (2)
C6—C1—C2—C3	−1.1 (2)	C17—C18—C19—C20	−4.4 (3)
C1—C2—C3—C4	43.3 (2)	C13—C18—C19—C20	177.93 (17)
C2—C3—C4—C5	−62.0 (2)	C18—C19—C20—C21	0.7 (3)
C3—C4—C5—C10	−136.41 (19)	C19—C20—C21—C22	2.3 (3)
C3—C4—C5—C6	39.9 (2)	C18—C17—C22—C21	−2.3 (3)
C10—C5—C6—C7	3.6 (3)	C16—C17—C22—C21	173.96 (19)
C4—C5—C6—C7	−172.80 (17)	C20—C21—C22—C17	−1.5 (3)
C10—C5—C6—C1	178.87 (17)	C6—C1—N1—N2	168.21 (15)
C4—C5—C6—C1	2.5 (3)	C2—C1—N1—N2	−10.7 (2)
N1—C1—C6—C7	−26.9 (3)	C6—C1—N1—Ni1	−14.6 (3)
C2—C1—C6—C7	151.95 (17)	C2—C1—N1—Ni1	166.55 (13)
N1—C1—C6—C5	157.95 (17)	N3—C11—N2—N1	174.85 (16)
C2—C1—C6—C5	−23.2 (2)	S1—C11—N2—N1	−4.2 (2)
C5—C6—C7—C8	−4.1 (3)	C1—N1—N2—C11	−154.97 (16)
C1—C6—C7—C8	−179.19 (17)	Ni1—N1—N2—C11	27.43 (18)
C6—C7—C8—C9	1.6 (3)	N2—C11—N3—C12	−5.6 (3)
C7—C8—C9—C10	1.4 (3)	S1—C11—N3—C12	173.59 (15)
C8—C9—C10—C5	−1.8 (3)	C18—C13—N4—N5	175.14 (15)
C6—C5—C10—C9	−0.7 (3)	C14—C13—N4—N5	−5.7 (2)
C4—C5—C10—C9	175.61 (18)	C18—C13—N4—Ni1	−9.9 (3)
N4—C13—C14—C15	178.27 (17)	C14—C13—N4—Ni1	169.25 (13)
C18—C13—C14—C15	−2.5 (2)	N6—C23—N5—N4	−178.50 (16)
C13—C14—C15—C16	45.5 (2)	S2—C23—N5—N4	0.6 (2)
C14—C15—C16—C17	−62.3 (2)	C13—N4—N5—C23	−157.81 (16)
C15—C16—C17—C22	−139.35 (19)	Ni1—N4—N5—C23	26.35 (18)
C15—C16—C17—C18	36.9 (2)	N5—C23—N6—C24	−5.7 (3)
C22—C17—C18—C19	5.1 (3)	S2—C23—N6—C24	175.10 (15)
C16—C17—C18—C19	−171.21 (17)	N2—C11—S1—Ni1	−16.07 (16)
C22—C17—C18—C13	−177.13 (17)	N3—C11—S1—Ni1	164.83 (14)
C16—C17—C18—C13	6.5 (3)	N5—C23—S2—Ni1	−22.12 (16)
N4—C13—C18—C19	−28.0 (3)	N6—C23—S2—Ni1	157.04 (14)
C14—C13—C18—C19	152.78 (18)	O1—C25—N7—C27	0.3 (3)
N4—C13—C18—C17	154.31 (17)	O1—C25—N7—C26	179.2 (2)

### Hydrogen-bond geometry (Å, º)

**Table:**

*D*—H···*A*	*D*—H	H···*A*	*D*···*A*	*D*—H···*A*
N3—H3···O1^i^	0.88	2.18	2.979 (2)	151
N6—H6···O1	0.88	2.14	2.875 (2)	140
C7—H7···Ni1	0.95	2.50	3.0831 (19)	120
C19—H19···Ni1	0.95	2.57	3.1480 (19)	120

Symmetry code: (i) −*x*+1, *y*−1/2, −*z*+1/2.
